# D-serine as a gliotransmitter and its roles in brain development and disease

**DOI:** 10.3389/fncel.2013.00039

**Published:** 2013-04-23

**Authors:** Marion R. Van Horn, Mari Sild, Edward S. Ruthazer

**Affiliations:** Montreal Neurological Institute, McGill UniversityMontreal, QC, Canada

**Keywords:** gliotransmission, D-serine, NMDA receptors, schizophrenia, depression and anxiety disorders, neural development, glycine site, astrocyte–neuron interactions

## Abstract

The development of new techniques to study glial cells has revealed that they are active participants in the development of functional neuronal circuits. Calcium imaging studies demonstrate that glial cells actively sense and respond to neuronal activity. Glial cells can produce and release neurotransmitter-like molecules, referred to as gliotransmitters, that can in turn influence the activity of neurons and other glia. One putative gliotransmitter, D-serine is believed to be an endogenous co-agonist for synaptic *N*-methyl-D-aspartate receptors (NMDARs), modulating synaptic transmission and plasticity mediated by this receptor. The observation that D-serine levels in the mammalian brain increase during early development, suggests a possible role for this gliotransmitter in normal brain development and circuit refinement. In this review we will examine the data that D-serine and its associated enzyme serine racemase are developmentally regulated. We will consider the evidence that D-serine is actively released by glial cells and examine the studies that have implicated D-serine as a critical player involved in regulating NMDAR-mediated synaptic transmission and neuronal migration during development. Furthermore, we will consider how dysregulation of D-serine may play an important role in the etiology of neurological and psychiatric diseases.

## INTRODUCTION

Two decades ago a revolutionary discovery identified the presence of the D-amino acid D-serine in mammalian brain. The discovery that D-serine is an abundant amino acid in the brains of rodents and humans led to extensive experimental inquiry into how this amino acid, which was previously thought to have no specific biological function, might be involved in normal brain development and function. In this review we will consider the development of analytical techniques that have been used to identify and localize D-serine within mammalian brains and review what is now known about its important biological function during brain development.

### D-SERINE LOCALIZATION WITHIN GLIA IN SPECIFIC BRAIN AREAS IN PROXIMITY TO NMDARs

**FIGURE 1:**
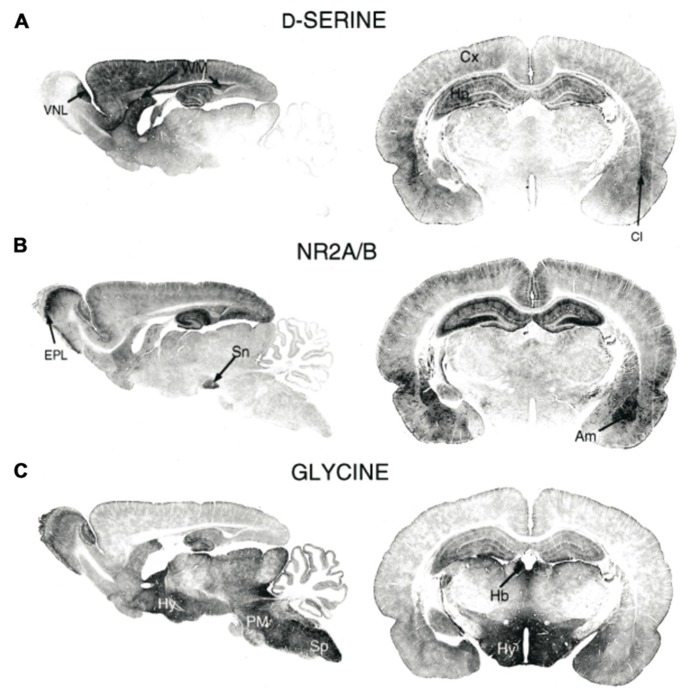
**Immunohistochemical staining of (A)** D-serine, **(B)** NMDAR subunits GluN2A/B, and **(C)** glycine in rat brains (P21). Am, amygdala; Cl, claustrum; Cx, cortex; EPL, external plexiform layer; Hb, habenula; Hy, hippocampus; PM, pons/medulla; Sn, substantia nigra; Sp, spinal cord; WM, white matter; VNL, vomeronasal nerve layer. Figure modified from Schell et al. (1997).

With the development of sensitive analytical techniques, such as gas chromatography and mass spectrometry, D-serine was discovered in mammalian brains about 20 years ago. In particular, Hashimoto et al. (1992) showed, using these two techniques, that D-serine was present in rodent and human brains at significantly higher concentrations than other D-amino acids, such as D-aspartate and D-alanine (Hashimoto et al., 1992, 1993a). Moreover, they showed that the distribution of D-serine paralleled the distribution of *N*-methyl-D-aspartate (NMDA) type glutamate receptors (Hashimoto et al., 1993a; **Figures 1A** and **B**). While previous work had determined that D-amino acids were capable of binding the glycine modulatory site of the NMDA receptor (Kleckner and Dingledine, 1988), the findings of Hashimoto et al. (1992, 1993a, b) pointed to D-serine as a potential endogenous co-ligand for the NMDA receptor. This role for D-serine as an endogenous NMDA receptor co-ligand helped explain the observation that glycine is not typically enriched in brain regions that have high levels of NMDA receptor expression (Schell et al., 1997; **Figure 1C**). Furthermore, D-amino acid oxidase (DAAO), the enzyme that degrades D-serine, had been discovered in mammals (Weimar and Neims, 1977; Horiike et al., 1987) before this demonstration of endogenous D-serine. Thus, with the discovery of D-serine, the ability of endogenous NMDA receptors to function in the absence of glycine and the presence of DAAO in the central nervous system (CNS) were both demystified.

Since the initial detection of D-serine in the CNS, subsequent immunostaining studies have more precisely localized D-serine and DAAO within specific brain areas and specific cell types. In the adult brain, areas with particularly high levels of NMDARs, including the cerebral cortex, hippocampus, thalamus, hypothalamus, amygdala, and retina are enriched in D-serine whereas other brain regions, such as the hindbrain, pons, and medulla have virtually undetectable levels of D-serine (Schell et al., 1995). Intriguingly, the localization of DAAO is opposite that of D-serine; areas including the cortex, which have high levels of D-serine have very low levels of DAAO.

After looking more closely at the distribution of D-serine amongst different cell types Schell et al. (1995) made the striking and important discovery that D-serine is localized principally within glial cells. Specifically, they found that type-2 astrocytes, cultured from cerebral cortex, expressed particularly high levels of D-serine. Additional studies in other brain areas have also localized D-serine within astrocytes. In the retina, Stevens et al. (2003) found D-serine in astrocytes and Müller glia cells. Furthermore, studies in the hippocampus, hypothalamus, vestibular nuclei (VN), and cerebellum have all placed the main expression of D-serine within glia cells (Mothet et al., 2000; Kim et al., 2005; Panatier et al., 2006; Puyal et al., 2006). Accumulating evidence, however, suggests that the synthesis, storage, and release of D-serine may not be limited exclusively to astrocytes, but rather may involve distinct functions for different cells.

### D-SERINE SYNTHESIS AND DEGRADATION

The key enzyme thought to be responsible for the synthesis of D-serine is serine racemase (SR). SR, which converts L-serine to D-serine, was initially found in astrocytes and microglia in the mammalian brain (Wolosker et al., 1999; Stevens et al., 2003; Wu et al., 2004; Panatier et al., 2006). More recently, however, SR was also identified in neurons (Kartvelishvily et al., 2006; Yoshikawa et al., 2007; Dun et al., 2008; Miya et al., 2008; Wolosker et al., 2008; Rosenberg et al., 2010) challenging the traditional view that D-serine is generated solely by astrocytes. In fact, SR was found to be expressed at significantly higher levels in neurons than in astrocytes. A study that compared SR and D-serine levels in cell type-specific SR knockout mice found that SR levels were lower in mice with neuronal knockout of SR than in mice with astrocyte-specific knockout of SR (Benneyworth et al., 2012). Surprisingly, despite a significant reduction of SR protein in neuronal SR knockout mouse brains, D-serine levels were only minimally reduced indicating that neurons are not the sole source of D-serine.

### REGULATION OF SR

A number of proteins that interact directly with SR have been identified, including the Golgin subfamily A member 3 protein (Dumin et al., 2006), the glutamate receptor interacting protein (GRIP; Kim et al., 2005), and the protein interacting with C-kinase 1 (PICK1; Fujii et al., 2006; Hikida et al., 2008). Furthermore, a more recent study showed that protein kinase C (PKC), which interacts directly with PICK1, is able to phosphorylate SR, leading to a corresponding decrease in D-serine levels *in vitro* and *in vivo* (Vargas-Lopes et al., 2011). There is also evidence suggesting that activation of erythropoietin-producing hepatocellular carcinoma (Eph) receptors decreases interaction between Eph–PICK1 and instead favors the SR–PICK1 interaction, resulting in a subsequent increase in D-serine synthesis in cultured hippocampal astrocytes (Zhuang et al., 2010).

### THE D-SERINE SHUTTLE HYPOTHESIS

In addition to catalyzing the conversion of L-serine to D-serine, SR can also cause the degradation of serine through the biochemical elimination of water, resulting instead in the production of pyruvate and ammonia (De Miranda et al., 2002). This degradation function of SR is likely to be important in regions of the brain, including the forebrain, that have with low levels of DAAO expression (Hashimoto et al., 1993b; Nagata et al., 1994). Astrocytes, having lower levels of SR compared to neurons, would be ideally suited for the safe storage of D-serine, effectively sequestering it from degradation by neuronal SR.

Interestingly, L-serine and its precursors are not abundant in neurons but found primarily in glial cells suggesting that although neurons have high levels of SR they require an external source of L-serine. For example, 3-phosphoglycerate dehydrogenase (Phgdh) an enzyme that catalyzes the formation of L-serine from glucose is localized almost exclusively in astrocytes (Furuya et al., 2000; Yamasaki et al., 2001) and a recent study has shown that a conditional deletion of Phgdh results in a significant decrease in both L- and D-serine levels in adult cerebral cortex and hippocampus (Yang et al., 2010). It has been suggested that the biosynthetic pathway for L-serine may be located in astrocytes but not neurons, requiring the transport of astrocytic L-serine to neurons where it can then be converted to D-serine for subsequent storage back in astrocytes.

**FIGURE 2:**
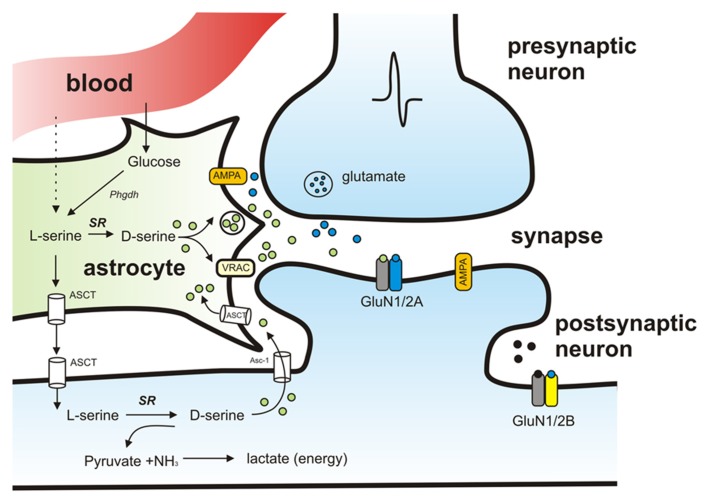
**Schematic model of the proposed pathways mediating D-serine synthesis and release.** Activation of presynaptic neuron results in release of glutamate that binds to AMPA receptors on neighboring astrocytes and causes release of D-serine. D-serine released from astrocytes binds to synaptic NMDAR-containing GluN2A subunits. Extrasynaptic receptors containing GluN2B preferentially bind glycine instead of D-serine. SR localized in neurons synthesizes D-serine from L-serine. L-serine is shuttled to neurons from astrocytes through amino acid transporters (ASCT). SR is also responsible for the degradation of serine resulting in production of pyruvate and ammonia.

Taken together there is accumulating evidence supporting a “D-serine shuttle hypothesis” which proposes that D-serine synthesized in neurons is shuttled to astrocytes where it is stored and released (Wolosker, 2011; **Figure 2**). Amino acid transporters have been identified in astrocytes and neurons (Yamamoto et al., 2004) and are thought to play an important role the transfer of amino acids between neurons and glia. Specifically, Na^+^-dependent ASCT1 and ASCT2 and Na^+^-independent alanine–serine–cystein transporter-1 (Asc-1) are two types of transporters that regulate D-serine levels. Of these, Asc-1, which is found exclusively in neurons, has a higher affinity for D-serine than ASCT1 and ASCT2 (Fukasawa et al., 2000; Helboe et al., 2003) and activation of Asc-1 by D-isoleucine has recently been shown to increase D-serine levels and to play a role in modulating synaptic plasticity (Rosenberg et al., 2013).

While it is generally agreed that astrocytic D-serine is necessary for normal glutamatergic transmission, the relative contribution of neuron- versus astrocyte-derived D-serine remains controversial and is likely to change over development and to differ by brain region.

### RELEASE OF D-SERINE

A number of studies have now clearly shown that the release of D-serine from astrocytes can be stimulated with the application of non-NMDA glutamate receptor agonists (Schell et al., 1995; Ribeiro et al., 2002; Mothet et al., 2005; Sullivan and Miller, 2010).

Using a sensitive chemoluminescence assay, Mothet et al. (2005) were able to demonstrate that D-serine release from cortical cultured astrocytes is evoked by glutatmate, α-amino-3-hydroxyl-5-methyl-4-isoxazole-propionate (AMPA) or kainic acid application, and is inhibited in the presence of AMPA blockers. The AMPA-evoked release of D-serine has been further supported by studies in other brain areas. Using capillary electrophoresis in the intact retina Sullivan and Miller (2010) have shown AMPA stimulates D-serine release and that release is abolished in the presence of a glial toxin. Furthermore, in primary glial cultures from cerebellum, activation of α-amino-3-hydroxyl-5-methyl-4-isoxazole-propionate receptors (AMPARs) has also been shown to trigger activation of SR by binding to GRIP to drive subsequent efflux of D-serine (Kim et al., 2005).

One important but somewhat contentious observation is the finding that release of D-serine from astrocytes may involve vesicular trafficking. Using the chemiluminescence assay for measuring D-serine, Mothet et al. (2005) found evidence that D-serine release is a SNARE (soluble NSF attachment protein receptor) protein-dependent process that requires calcium influx. D-serine release from astrocytes is impaired by applying tetanus toxin light chain, an endopeptidase that cleaves SNARE proteins necessary for vesicular fusion (Martineau et al., 2008). Furthermore, more recent studies in hippocampal slices have provided additional evidence that at least some of astrocytic D-serine release relies on vesicle-associated membrane protein (VAMP)-dependent exocytosis (Henneberger et al., 2010). In contrast, Rosenberg et al. (2010) showed that activation of volume-regulated anion channels (VRACs) resulted in a significant amount of D-serine released from astrocytic cultures, but that blocking vesicular filling using bafilomycin and concanamycin, which are inhibitors of vacuolar ATPase, did not affect D-serine release. Notably, Rosenberg et al. (2010) highlighted the fact that while both neurons and astrocytes can release D-serine, the mechanisms mediating D-serine release from neurons are likely to be different from those in astrocytes. They found that depolarization of neurons in cortical slices using veratridine, which induced D-serine release by neurons but not by astrocytes, resulted in 10-fold greater levels of D-serine release than AMPA receptor activation, which is thought to evoke release from astrocytes.

A recent series of studies has provided new lines of evidence that astrocytes and neurons can release D-serine through different mechanisms. In particular, a detailed analysis of the contents of rat cortical astrocytes has revealed that these cells contain storage vesicles that share features similar to synaptic vesicles. Moreover, they found that cortical astrocytic vesicles contain high levels of D-serine, in addition to other neuromodulators including glutamate and glycine (Martineau et al., 2013). In contrast, Rosenberg et al. (2013) has used both cultured cells and acute hippocampal slices to demonstrate that Asc-1 mediates release of D-serine from cytosolic pools in neurons.

Additional studies are still needed to determine the relative importance and function of astrocytic versus neuronal D-serine. To date, much of what is known about D-serine release comes from *in vitro* studies but it will become increasingly important to accurately measure D-serine concentrations *in vivo*. For example, *in vivo* microdialysis has been used to measure extracellular concentrations of D-serine in mouse barrel cortex (Takata et al., 2011). It was found that stimulation of the nucleus basalis of Meynert resulted in an increase in D-serine concentration that could not be induced in inositol-1,4,5-trisphosphate receptor type 2 knockout mice, in which astrocytic calcium fluctuations are reduced.

Recently, implantable amperometric biosensors have been developed which will be extremely valuable for monitoring *in vivo* release of D-serine (Pernot et al., 2008, 2012; Zain et al., 2010). These biosensors consist of a thin platinum wire coated with a semi-permeable layer of poly-*m*-phenylene-diamine (PPD) and a layer of DAAO. Degradation of available D-serine is catalyzed by DAAO, generating hydrogen peroxide that is oxidized at the surface of the platinum wire. The resulting hydrogen peroxide oxidation current corresponds to the D-serine concentration in the surrounding environment. To date, biosensors implanted in the rat brain have been used to detect D-serine *in vivo* (Zain et al., 2010; Pernot et al., 2012). Alternatively, the temporal resolution of *in vivo* microdialysis has been recently improved to the point that it is now possible to detect D-serine levels with a resolution of several seconds (Rosenberg et al., 2010). Additional studies determining what factors influence and modulate D-serine levels *in vivo* are sure to follow shortly.

### D-SERINE IS A CRITICAL PLAYER IN NMDAR-MEDIATED SYNAPTIC PLASTICITY

NMDARs are unique in that they require the binding of two agonists to be fully functional. In particular, activation of NMDARs requires the binding of glutamate to the GluN2 subunit as well as the binding of a separate co-agonist to the glycine site of the GluN1 subunit (Johnson and Ascher, 1987). While D-serine was originally identified as an effective co-agonist of the NMDAR (Kleckner and Dingledine, 1988), glycine was thought to be a more likely endogenous candidate because, at the time, more was known about its presence in mammalian brain. In light of the considerable accumulation of evidence that D-serine is indeed present in many mammalian brain areas, D-serine has come to be accepted as an endogenous co-agonist for synaptic NMDARs, involved in modulating synaptic transmission and plasticity. Further support for this idea came from a pioneering study that monitored NMDAR-mediated synaptic transmission in hippocampal cell cultures after the selective degradation of endogenous D-serine by application of DAAO (Mothet et al., 2000). It was found that when cells were exposed to DAAO, the NMDAR currents were significantly reduced and this reduction could be reversed with application of exogenous D-serine. More recent evidence from Papouin et al. (2012) implicates D-serine as the endogenous co-agonist specifically at synaptic NMDARs in the hippocampus.

As NMDARs are known to be a key player in mediating excitatory transmission and synaptic plasticity, such as long-term potentiation (LTP; Constantine-Paton et al., 1990), astrocyte-derived D-serine is also likely to play an important modulatory role. Yang et al. (2003) designed an elegant experiment to address this question *in vitro*. Specifically, the ability to evoke LTP in cultured neurons was compared between cells grown in direct contact with astrocytes and those grown without direct contact. Strikingly, they found that LTP could not be induced in the neurons that were not in direct contact with astrocytes unless the cells were supplemented with an exogenous source of D-serine (Yang et al., 2003). The important contribution of D-serine to activity-induced synaptic plasticity has since been further confirmed in other brain areas including the hypothalamus, retina, and prefrontal cortex (Panatier et al., 2006; Henneberger et al., 2010; Stevens et al., 2010; Fossat et al., 2012).

In the hypothalamus, Panatier et al. (2006) took advantage of the fact that the physical association of astrocytes with neurons in the supraoptic nucleus (SON) changes during pregnancy. In particular, during lactation the release of the hormone oxytocin causes a retraction of astrocytic processes from synaptic sites in the SON, which results in a decrease in available D-serine at the synaptic cleft. Panatier et al. (2006) found a corresponding decrease in NMDAR-mediated synaptic activity as well as an increase in the threshold required to induce LTP in the SON.

More recently, Henneberger et al. (2010) have extended these findings by providing evidence that the release of D-serine from astrocytes in the hippocampus is likely to be a calcium-dependent process and that an individual astrocyte can contribute to NMDAR-dependent plasticity in many surrounding neuronal synapses. Specifically, using a technique they developed called “calcium-clamping” where intracellular astrocytic calcium was chemically clamped at a fixed concentration, and therefore unable to fluctuate rapidly, they were able to show that LTP could no longer be induced in neighboring neurons unless exogenous D-serine was supplemented. A similar result was obtained by loading astrocytes with tetanus toxin to prevent VAMP-dependent vesicular release. Notably, they also showed that one astrocyte in the hippocampus appeared to be responsible for a specific territory of neighboring neurons and that neurons found outside of this specific area (i.e., >200 μm) were unaffected by these manipulations. These specific findings were somewhat surprising since the anatomical territory of an astrocyte in the hippocampus is generally considered to be only about 50–100 μm large, raising the possibility that communication between adjacent astrocytes may participate in this phenomenon.

An interesting feature of NMDARs is that they are found both synaptically and extrasynaptically. The NMDAR has been shown to have different physiological functions depending on its location. While NMDARs found at synapses have a well-established role in mediating synaptic LTP, extrasynaptic NMDARs have been implicated in other forms of plasticity, as well as in the pathogenesis of certain neurodegenerative diseases including Huntington’s and Alzheimer’s disease (Kaufman et al., 2012).

Recent results have shed some light as to why synaptic versus extrasynaptic NMDARs may have distinct physiological functions. Papouin et al. (2012) have shown that synaptic and extrasynaptic NMDARs in the hippocampus preferentially bind different co-agonists. In particular, by applying enzymes to selectively degrade either D-serine or glycine they were able to demonstrate that D-serine preferentially affects synaptic NMDARs whereas glycine preferentially affects extrasynaptic receptors. When D-serine was degraded using DAAO, synaptically mediated NMDAR functions, including LTP, were abolished. Conversely, the application of glycine oxidase, an enzyme that specifically degrades glycine, had no effect on synaptic excitatory potentials.

*N*-methyl-D-aspartate receptor affinity for binding D-serine versus glycine depends on its GluN2 subunit composition. While NMDARs composed of GluN2A subunits exhibit a high affinity for D-serine, NMDARs composed of GluN2B subunits preferentially bind glycine (Madry et al., 2007). Papouin et al. (2012) took advantage of the existence of antagonists with preferential affinities for GluN2A and GluN2B to further determine the respective contributions of D-serine and glycine in regulating synaptic versus extrasynaptic NMDAR activation. They found that NMDAR-mediated post-synaptic potentials were reduced in the presence of free Zn, an antagonist of GluN2A-containing receptors, but were unaffected in the presence of Ro25-6981, a selective GluN2B antagonist. To study the endogenous co-agonist of extrasynaptic NMDARs, synaptic receptors were silenced using MK801. Under synaptic NMDAR blockade, the spared extrasynaptic NMDA-evoked responses were decreased when glycine was specifically degraded. These results indicate that that while D-serine is an important co-agonist of synaptic NMDARs, glycine may function as the principal partner for extrasynaptic receptors.

### DEVELOPMENTAL EXPRESSION OF D-SERINE AND THE DEVELOPMENT OF GLUTAMATERGIC SYNAPSES

During early development neurons undergo extensive synaptic refinement and maturation as they establish functional neuronal circuits. It has been well-established that NMDARs play a critical role in the development of neuronal circuits. Blocking their activity has been shown in many cases to disrupt normal activity-dependent map formation (Constantine-Paton et al., 1990; Ruthazer and Cline, 2004; Espinosa et al., 2009; Erzurumlu and Gaspar, 2012).

Immunohistochemical localization studies of D-serine in rat brain have illustrated that D-serine levels change over different stages of development. For example, the VN, an area specialized in controlling balance and spatial orientation, have high levels of D-serine during the first 3 weeks of post-natal development, which gradually decrease with age (Puyal et al., 2006). Furthermore, in the cerebellum, a brain region important for motor coordination and learning, D-serine levels are high during the first weeks after birth but then rapidly decline to almost undetectable levels by P26. As in other brain areas, D-serine has been localized within specific glia cells bodies of the cerebellum. In particular, high levels of D-serine are found in the radial processes and end feet of Bergmann glia surrounding Purkinje cell dendrites (Schell et al., 1997; Wolosker et al., 1999). Notably, the drop in D-serine levels parallels the increase in expression of DAAO in cerebellum (Weimar and Neims, 1977; Horiike et al., 1987).

During early post-natal development the cerebellum undergoes a series of important developmental changes that are essential for forming a functional cerebellar circuit. It is a time when parallel and climbing fibers form stereotypic synaptic connections with Purkinje cells. This establishment of functional synapses has been shown to be dependent on NMDARs since blocking the receptors prevents the formation of proper synapses (Rabacchi et al., 1992). Given the tight physical interaction between Bergmann glia and Purkinje cells it has been proposed that D-serine release from Bergmann glia may be an important player in the development of functional synapses in the cerebellum (Schell et al., 1997), however, the specific involvement of D-serine in establishing connections in the cerebellum remains to be examined. Consistent with the idea that D-serine may play a role in promoting the formation of functional synapses is the recent evidence that synaptogenesis induced by transforming growth factor β-1 (TGF-β1) is dependent on D-serine (Packard et al., 2003; Diniz et al., 2012). In cultured neurons Diniz et al. (2012) have shown that TGF-β1 secreted from astrocytes promotes the NMDAR-dependent formation of synapses. Interestingly, in addition to inducing synaptogenesis, TGF-β1 also results in an increase in extracellular levels of D-serine. Furthermore, application of D-serine alone was sufficient to induce the formation of synapses, similar to TGF-β1 treatment. The synaptogenic property of TGF-ß1 was eliminated when D-serine was reduced by DAAO treatment, suggesting that D-serine release is responsible for TGF-β1-mediated synaptogenesis.

The development and refinement of functional neuronal circuits is mediated, at least in part, by neurotrophins. In particular, brain-derived neurotrophic factor (BDNF) has been found to be an activity-regulated neurotrophin that plays a key role in promoting synapse formation and maturation and in regulating the functional development of neuronal circuits (Huang et al., 1999; Kaneko et al., 2008; Cohen-Cory et al., 2010; Schwartz et al., 2011; Park and Poo, 2012). Consistent with evidence that NMDAR activation can regulate BDNF production, a recent study found that SR knockout mice (SR^-^^/^^-^) have a reduction in total BDNF protein levels (Balu et al., 2012). This result further underscores the importance of D-serine as a potential mediator in the establishment of precise neuronal connections during development.

The subunit composition of NMDARs changes over development and by brain region, suggesting that NMDAR subtypes may play different developmental roles. In the hippocampus GluN2B expression peaks early in development while GluN2A expression occurs later. This shift in GluN2A/2B subunit expression has been correlated with the maturation of neuronal circuits and the control of a number of important developmental events (van Zundert et al., 2004; Yashiro and Philpot, 2008). For example, in the rodent visual cortex the developmental switch from GluN2B to GluN2A is delayed by extended dark-rearing (Quinlan et al., 1999).

Recent evidence has shown that the availability of D-serine and glycine preferentially influences the diffusion of NR2B versus NR2A subunits (Burnet et al., 2011; Papouin et al., 2012), suggesting that D-serine may play a role in the development of neuronal circuits potentially by influencing the subunit make-up of glutamatergic synapses. Experiments by Papouin et al. (2012) revealed that, increasing the amounts of D-serine or glycine selectively inhibited surface trafficking of GluN2B and GluN2A, respectively. The mechanisms by which D-serine and glycine inhibit the diffusion of GluN2A or 2B are unknown, however, conformational changes in the receptor have been proposed as a means by which diffusion could be prevented. Furthermore, a DAAO knockdown in mouse cerebellum, resulting in elevated D-serine levels has been shown to lead to a decrease in GluN2A mRNA levels (Burnet et al., 2011). These experiments reveal that D-serine and glycine exert a surprising degree of control over functional NMDAR subunit composition at multiple levels, ranging from synthesis to trafficking to activation.

### D-SERINE AND THE DEVELOPMENT OF DENDRITIC MORPHOLOGY

NMDARs are required for the proper development of dendritic arbors. NMDAR blockade in the developing brain results in a drastic decrease in dendritic growth (Rajan and Cline, 1998; Sin et al., 2002). Recently, the importance of D-serine in mediating normal dendritic development has been examined in SR^-^^/^^-^ mice (DeVito et al., 2011; Balu and Coyle, 2012). The dendrites of pyramidal neurons in prefrontal cortex, as well as primary somatosensory cortex, of SR^-^^/^^-^ mice are less complex and have a shorter total length (Balu et al., 2012). SR^-^^/^^-^ animals have a small but significantly reduced cortical volume. As expected, the dendritic arbor changes were not as severe when neuronal SR was deleted in older mice after weaning, indicating that there is an early post-natal critical period in which SR is most effective in affecting dendritic development. The relative contributions of neuronal and astrocytic SR to normal dendritic development and maintenance remain to be fully explored.

### NEURONAL MIGRATION

In addition to its important role in the formation and maturation of synaptic contacts, D-serine has been demonstrated to contribute to earlier stages neuronal circuit construction, as a regulator of neuroblast migration in the developing brain. High levels of D-serine have been localized in the radial processes and end feet of Bergmann glia surrounding Purkinje cell dendrites (Schell et al., 1997; Wolosker et al., 1999). During the period of early post-natal development the cerebellum undergoes a series of important developmental changes that are essential for forming a functional cerebellar circuit. Granule cells, the primary excitatory neurons in the cerebellar circuit, migrate from the external to internal granule cell layer and this migration processes has been shown to occur along radial glial cells which give rise to Bergmann glia.

The completion of granule cell migration has been found to correspond to decreasing levels of D-serine suggesting that D-serine may be an important player in the granule cell migration process. Indeed, Kim et al. (2005) found that when D-serine was degraded by DAAO this resulted in a reduction in neuronal migration by ~60%, which could be reversed with application of exogenous D-serine. Furthermore, inhibition of SR also was found to result in a reduction in migration. Overall, the evidence suggests that D-serine acts as chemokinetic stimulus to granule cells (Kim et al., 2005).

The cerebellum is a clear and well-studied example of a brain area where neurons migrate along glia to reach their mature position in an organized neuronal circuit. However, radial cell migration is not limited to the cerebellum and thus it will be interesting to further determine how D-serine may be involved in neuronal migration in other brain areas that depend on directed migration, such as neocortex.

## D-SERINE AND DISORDERS OF GLUTAMINERGIC FUNCTION

D-serine has emerged as an influential player in the context of psychiatric disease. Motivated in large part by the hypothesis that schizophrenia and depression may represent dysregulation of glutamatergic systems, with a particular emphasis on NMDAR transmission (Carlsson and Carlsson, 1990; Bennett, 2009; Gunduz-Bruce, 2009), there has been an increasing number of studies exploring D-serine, both endogenously as a potential cause and exogenously as a potential therapy for these disease conditions.

### SCHIZOPHRENIA

Schizophrenia is characterized by disintegration of thought processes and emotional responsiveness (Lewis and Lieberman, 2000; van Os and Kapur, 2009). The etiology of schizophrenia is believed to involve a combination of genetic and environmental factors. Schizophrenia symptoms can be categorized as positive and negative: positive symptoms include delusions and hallucinations whereas negative symptoms include anhedonia, asociality, alogia, avolition, and blunted emotional response. In addition, schizophrenia patients suffer from varying degrees of cognitive deficit. A model of glutaminergic hypofunction with accompanying disinhibition of the dopaminergic system has gained significant support (Carlsson and Carlsson, 1990; Lisman et al., 2008; Bennett, 2009; Gunduz-Bruce, 2009). This hypothesis, which proposes that NMDAR hypofunction may be responsible in part for schizophrenia, is premised upon the observation that NMDAR antagonists like phencyclidine (PCP) and ketamine are able to induce a wide spectrum of schizophrenia-like symptoms in normal human subjects (Javitt and Zukin, 1991; Krystal et al., 1994; Coyle, 1996; Olney et al., 1999). This hypothesis initially led to the use of D-serine as a potential therapeutic for alleviating schizophrenia (Contreras, 1990). Subsequently, a deficiency in endogenous D-serine availability was proposed as a potential underlying cause of NMDAR hypofunction.

Consistent with this hypothesis, several studies have revealed a reduction in D-serine levels in the plasma and cerebrospinal fluid of schizophrenic patients (Hashimoto et al., 2003; Bendikov et al., 2007; Calcia et al., 2012). This may be explained in part by excessive degradation of D-serine, as supported by observations that DAAO levels and activity have been found to be elevated in post-mortem hippocampus and cortex samples from schizophrenia patients (Madeira et al., 2008; Habl et al., 2009).

Association studies have identified several mutations in human D-serine metabolic enzymes as risk factors for schizophrenia. These include single-nucleotide polymorphism (SNP) variants of SR (Morita et al., 2007), DAAO (Boks et al., 2007; Caldinelli et al., 2013), and the DAAO interacting protein G72 (Muller et al., 2011). Notably, the schizophrenia and depression susceptibility gene Disrupted in schizophrenia-1 (DISC1) appears to directly bind SR, protecting it from ubiquitin-mediated degradation (Ma et al., 2012).

D-serine, as an NMDAR co-agonist, has been tested as a potential therapeutic agent for schizophrenia. D-serine, in combination with antipsychotics, was reported to be more efficient in relieving numerous symptoms of schizophrenia than antipsychotics on their own (Tsai et al., 1998; Heresco-Levy et al., 2005; Kantrowitz et al., 2010). In mice, D-serine successfully reversed schizophrenia-like symptoms, such as the reduction in prepulse inhibition and certain cognitive deficits, caused by administration of NMDAR antagonists (Lipina et al., 2005; Hashimoto et al., 2008; Kanahara et al., 2008). However, chronic administration of D-serine can result in desensitization as well as kidney damage in patients. Thus, a number of alternative NMDAR glycine site agonists have been tested. The antibiotic D-cycloserine and a synthetic amidated tetrapeptide GLYX-13 are both partial agonists of the NMDAR glycine site and therefore do not function identically to the full agonist D-serine (Kanahara et al., 2008; Zhang et al., 2008). The assessment of D-cycloserine effects in schizophrenia patients has not been straightforward (Duncan et al., 2004). It has been proposed that D-cycloserine may only be efficient when used in treatment together with a training paradigm (Gottlieb et al., 2011). Glycine, also a full agonist at the NMDAR glycine site, has been reported to relieve negative and positive schizophrenia symptoms in patients and to do so more efficiently than D-cycloserine (Heresco-Levy and Javitt, 2004). A meta-analysis reviewing a multitude of trials with schizophrenia patients receiving D-serine, glycine and D-cycloserine came to the conclusion that the full agonists were more effective at relieving negative symptoms of schizophrenia than D-cycloserine, but that no glutaminergic therapy successfully attenuated the positive symptoms (Tuominen et al., 2005).

### DEPRESSION AND D-SERINE

The etiology of mood disorders [major depressive disorder (MDD), bipolar disorder and dysthymic disorder] is also not well-understood (Hidaka, 2012). MDD constitutes a disturbance of mood accompanied by a selection of psychophysiological changes, such as slowing of speech and action, insomnia or hypersomnia, fatigue, psychomotor agitation or retardation, and loss or increase of appetite (Cassano and Fava, 2002; Belmaker and Agam, 2008). Common psychological symptoms include loss of concentration, perfectionism/obsessiveness, increased sensitivity to criticism, feelings of worthlessness, anhedonia, and suicidal thoughts (Cassano and Fava, 2002). MDD is currently one of the leading causes of disability worldwide (Hidaka, 2012). MDD is generally considered to reflect a malfunction of the monoaminergic systems, but growing evidence also points toward the involvement of excitatory amino acids (Kugaya and Sanacora, 2005). Altered, usually elevated, levels of glutamate metabolites have been detected in post-mortem human prefrontal cortex (Hashimoto et al., 2007) and blood serum (Mauri et al., 1998). A proton magnetic spectroscopy study, which enabled quantification of glutamate and GABA levels in live depression patients, also revealed higher than normal glutamate concentrations in the prefrontal cortices of MDD patients, together with an abnormal inhibitory/excitatory neurotransmitter ratio (Sanacora et al., 2004).

Chronically stressed animals, considered to be a model for depression, exhibit profound changes in neuroplasticity, specifically impairment in hippocampal and prefrontal cortical LTP induction, and a facilitation of spike-timing dependent long-term depression (Shors et al., 1990; Shors, 2004; Pittenger and Duman, 2008; Marsden, 2013). Chronic restraint stress was shown to result in NMDAR-dependent dendritic atrophy in rat medial prefrontal cortex, that could be prevented by CPP administration (Martin and Wellman, 2011). The evidence for NMDAR involvement in animal depression models inspired the testing of NMDAR binding compounds. MK-801, AP-7, and 1-aminocyclopropanecarboxylic acid (ACPC) were all found to be as effective as common antidepressants in increasing the performance of mice in stress paradigms like the forced swim and the tail suspension test (Trullas and Skolnick, 1990). Ketamine has been repeatedly shown to relieve depression and anxiety in about 70% of human patients (Sappington et al., 1979; Zarate et al., 2006; Diazgranados et al., 2010).

Interestingly, whereas ketamine, MK-801 and AP-7 are all NMDAR antagonists, ACPC is a partial agonist for the NMDAR glycine site. Subsequently D-serine was tested on the so-called “Flinders Sensitive rat” strain that displays symptoms similar to human depression patients, for example reduced interest in reward and increased fear response (Overstreet et al., 2005). This rodent model for depression exhibits increased hippocampal glutamatergic activity. Its hippocampi appear to contain lower than normal levels of D-serine, as measured by enantioselective chromatography, perhaps due to a homeostatic compensation for the elevated glutamate, and are impaired for CA1 LTP induction. Flinders Sensitive rats were found to have worse object-recognition memory, which could be rescued by an acute injection of D-serine (Gomez-Galan et al., 2012). Similar benefits of acute D-serine injection were demonstrated in the Wistar Kyoto rat, another stress-sensitive animal model for depression studies (Malkesman et al., 2012). Importantly in this latter study, NMDARs in the forebrain were confirmed as the site of action of the D-serine treatment by demonstrating that rescue was not effective in forebrain targeted NR1 knockout mice. While it is difficult to distinguish between a specific requirement for NMDAR transmission and general excitatory transmission, such results provide support for the idea that disrupted NMDAR function may be an underlying factor in depression.

### COMMON SENSITIVITY TO D-SERINE IN SCHIZOPHRENIA AND DEPRESSION

It is intriguing to note that while depression is thought to constitute a hyperglutaminergic condition, relieved by NMDAR antagonists, schizophrenia is seen as a hypoglutaminergic disease worsened by NMDAR antagonists. Nonetheless, D-serine appears to mitigate both of those disorders. Similarly, whereas low plasma and cerebrospinal fluid (CSF) levels of D-serine are reported in schizophrenic patients, the Flinders Sensitive rat model for depression also exhibited lower than normal levels of D-serine in hippocampus. In the latter case, this was speculated to be the consequence of a protective downregulation of D-serine to spare neurons from excitotoxic effects in this hyperglutamatergic strain (Gomez-Galan et al., 2012). Binding of the glycine site on the NMDAR has been demonstrated to reduce NMDAR desensitization (Mayer et al., 1989). Thus, one possible way to reconcile the paradox of D-serine’s dual efficacy in schizophrenia and depression would be if the effects of hyperglutaminergia were in part mediated by NMDAR desensitization, with D-serine both attenuating desensitization and enhancing neurotransmission.

It is also likely that despite similar outcomes of helping restore more normal functional states, the major therapeutic targets of D-serine in schizophrenia and depression are different or at distinct locations. For example, the main site of dendritic atrophy in post-mortem samples from schizophrenia patients is reportedly the prefrontal cortex (Garey et al., 1998; Glantz and Lewis, 2000; Broadbelt et al., 2002; Garey, 2010), whereas in chronically stressed animals, spine and dendrite loss is mostly observed in hippocampus (Magarinos and McEwen, 1995; Magarinos et al., 1996; Luo and Tan, 2001; Chen et al., 2008; Bedrosian et al., 2011) and in some cases in prefrontal cortex (Cook and Wellman, 2004; Martin and Wellman, 2011). Hippocampal neuropil damage is supported by magnetic resonance imaging (MRI) studies in humans suffering from depression (Sheline et al., 1999; von Gunten et al., 2000; Stockmeier et al., 2004; MacQueen et al., 2008). Based on the important contributions of D-serine to normal dendrite formation during development (DeVito et al., 2011; Balu and Coyle, 2012; Balu et al., 2012), it offers great promise as a potential means for helping restore normal dendritic structure and function in schizophrenia and depression, albeit in heterogenous brain areas.

### APPLICATIONS OF D-SERINE THERAPY IN OTHER DISEASES

Due to the success of D-serine treatment in relieving symptoms of schizophrenia, its use has been extended to treat a variety of diseases. NMDAR antagonists have been effective in ameliorating Parkinsonian symptoms in animal models (Johnson et al., 2009). A pilot study involving 10 Parkinson disease patients treated with 30 mg/kg D-serine for 6 weeks in addition to their usual medications demonstrated improvements in motor and behavioral deficits (Gelfin et al., 2012). Using an identical treatment regimen, the same group reported anxiolytic properties for D-serine in a group of 22 post-traumatic stress disorder patients (Heresco-Levy et al., 2009). D-cycloserine has been found to have anxiolytic properties in patient cohorts with different phobias or obsessive-compulsive disorder. Interestingly, D-cycloserine seems to be effective only when combined with exposure therapy involving the object of fear or obsessiveness (Kushner et al., 2007; Guastella et al., 2008). Finally, autism has also been viewed by some as a hypoglutaminergic disorder (Carlsson, 1998). In concordance with that idea, high doses of both D-serine and D-cycloserine were found to improve the sociability of an inbred Balb/C mouse strain that exhibits autism-like behaviors (Jacome et al., 2011; Deutsch et al., 2012). The pre-clinical glycine site partial agonist GLYX-13 likewise has been reported to reverse autism-like symptoms in selectively bred non-social rats (Moskal et al., 2011).

### NEURODEGENERATIVE DISORDERS: THE DARK SIDE OF D-SERINE

In addition to alleviating several diseases and conditions, D-serine may have its dark side in neurodegenerative disease. Amyloid beta (Aβ) and secreted amyloid precursor protein appear to induce the release of D-serine and glutamate from cultured microglia by increasing transcription and stabilization of the microglial SR (Wu et al., 2004). This could contribute to excitotoxic neuronal death in Alzheimer’s disease. D-serine may play a similar role in amyotrophic lateral sclerosis (ALS) where a principal cause for motoneuron death is considered to be excitotoxicity (Bruijn et al., 2004). The cerebrospinal fluid of ALS patients has been reported to contain elevated glutamate levels (Rothstein et al., 1990). Interestingly, it has been observed that spinal cord in the G93A-SOD1 transgenic mouse model for ALS has several-fold higher concentrations of D-serine than control animals (Sasabe et al., 2007). In these mice, D-serine was found to be produced by activated microglia. The presence of endogenous D-serine has been shown to be necessary for most, if not all, NMDAR-mediated excitotoxicity observed in rat brain slices and *in vivo* (Katsuki et al., 2004; Hama et al., 2006; Inoue et al., 2008). Pretreatment of rat hippocampal organotypic slices with D-serine deaminase was shown to practically abolish NMDA-mediated excitotoxicity, suggesting that D-serine, rather than glycine, participates in these excitotoxic events in the hippocampus (Shleper et al., 2005). Furthermore, SR knockout mice exhibited a 50–60% reduction in the volume of brain damaged in a transient cerebral artery occlusion-induced stroke model. These SR knockout mice were protected against focal cerebral ischemia despite a fourfold increase in NR1 subunits and elevated NMDAR sensitivity (Mustafa et al., 2010). D-serine has also been hypothesized to participate in epileptogenesis. In animals with pilocarpine-induced epilepsy increased D-serine content in astroglia or neurons, but interestingly not microglia, has been reported (Liu et al., 2009).

## SUMMARY

Once thought to have no neurological significance, D-serine is now recognized as an important player in normal brain development and function. D-serine is localized in areas of the brain that have high NMDAR expression and is considered to be an important endogenous co-agonist of NMDARs in many brain regions, including the forebrain and hippocampus. D-serine regulates NMDAR-mediated synaptic transmission and plasticity. It has also been shown to be a key mediator in neuronal migration in the cerebellum. Just as our understanding of the various roles for D-serine in brain development has grown more sophisticated, our comprehension of the mechanisms underlying D-serine synthesis and release has also evolved. It has been proposed that neurons, which contain high SR, may play an important role in synthesizing D-serine while glial cells appear to play a more important role in its release (**Figure 2**). Still, our understanding of the specific roles of neuronal versus glial D-serine remain incomplete. Progress has been made using *in vitro* models, but the key issue of how signaling events influence and modulate D-serine levels *in vivo *is still an open question.**Overall, there is a great deal of accumulated evidence implicating D-serine as an important player in neuronal development, however, its specific roles in the refinement of functional neuronal circuits remains to be fully explored.

In light of its critical role in the normal development of neuronal circuits, it is hardly surprising that D-serine also participates in adult psychiatric health. D-serine regulation has been investigated extensively as a causative factor and in some cases as a potential therapeutic in schizophrenia, as well as a broad spectrum of other neurological disorders. Curiously, it appears to be beneficial for both NMDAR hypofunction (schizophrenia) and hyperfunction (depression) disease models. The explanation for this might lie in its differential effects on neuronal and glial sub-populations or in the particular brain regions impacted. It is tempting to speculate based on the important roles played by D-serine in the developing brain that its ability to remediate disease may in part depend on enhancing functional connectivity by supporting NMDAR-dependent synaptic plasticity, dendritic arborization, and synaptic transmission in the mature brain.

## Conflict of Interest Statement

The authors declare that the research was conducted in the absence of any commercial or financial relationships that could be construed as a potential conflict of interest.
